# Constitutive Cell Proliferation Regulating Inhibitor of Protein Phosphatase 2A (CIP2A) Mediates Drug Resistance to Erlotinib in an EGFR Activating Mutated NSCLC Cell Line

**DOI:** 10.3390/cells10040716

**Published:** 2021-03-24

**Authors:** Hisham Saafan, Ahmad Alahdab, Robin Michelet, Linus Gohlke, Janine Ziemann, Stefan Holdenrieder, Katie-May McLaughlin, Mark N. Wass, Jindrich Cinatl, Martin Michaelis, Charlotte Kloft, Christoph A Ritter

**Affiliations:** 1 Institute of Pharmacy, Clinical Pharmacy, University of Greifswald, Friedrich-Ludwig-Jahn-Str. 17, 17489 Greifswald, Germany; hisham.saafan@uni-greifswald.de (H.S.); ahmad.alahdab@uni-greifswald.de (A.A.); linus.gohlke@uni-greifswald.de (L.G.); 2 Department of Clinical Pharmacy and Biochemistry, Institute of Pharmacy, Freie Universitaet Berlin, 14195 Berlin, Germany; robin.michelet@fu-berlin.de (R.M.); charlotte.kloft@fu-berlin.de (C.K.); 3 Central Unit for Infection Prevention and Control, University Medicine Greifswald, 17475 Greifswald, Germany; janine.ziemann@uni-greifswald.de; 4 Institute of Laboratory Medicine, German Heart Center, Munich Technical University, 80636 Munich, Germany; stefan.holdenrieder@uni-bonn.de; 5 Industrial Biotechnology Centre, School of Biosciences, University of Kent, Canterbury CT2 7NJ, UK; km625@kent.ac.uk (K.-M.M.); m.n.wass@kent.ac.uk (M.N.W.); m.michaelis@kent.ac.uk (M.M.); 6 Institute of Medical Virology, Goethe-University, 60596 Frankfurt am Main, Germany; cinatl@em.uni-frankfurt.de

**Keywords:** non-small cell lung cancer, epidermal growth factor receptor, drug resistance, cell proliferation regulating inhibitor of protein phosphatase 2A, bortezomib

## Abstract

Exploring mechanisms of drug resistance to targeted small molecule drugs is critical for an extended clinical benefit in the treatment of non-small cell lung cancer (NSCLC) patients carrying activating epidermal growth factor receptor (EGFR) mutations. Here, we identified constitutive cell proliferation regulating inhibitor of protein phosphatase 2A (CIP2A) in the HCC4006rErlo0.5 NSCLC cell line adapted to erlotinib as a model of acquired drug resistance. Constitutive CIP2A resulted in a constitutive activation of Akt signaling. The proteasome inhibitor bortezomib was able to reduce CIP2A levels, which resulted in an activation of protein phosphatase 2A and deactivation of Akt. Combination experiments with erlotinib and bortezomib revealed a lack of interaction between the two drugs. However, the effect size of bortezomib was higher in HCC4006rErlo0.5, compared to the erlotinib-sensitive HCC4006 cells, as indicated by an increase in Emax (0.911 (95%CI 0.867–0.954) vs. 0.585 (95%CI 0.568–0.622), respectively) and decrease in EC50 (52.4 µM (95%CI 46.1–58.8 µM) vs. 73.0 µM (95%CI 60.4–111 µM), respectively) in the concentration–effect model, an earlier onset of cell death induction, and a reduced colony surviving fraction (0.38 ± 0.18 vs. 0.95 ± 0.25, respectively, *n* = 3, *p* < 0.05). Therefore, modulation of CIP2A with bortezomib could be an interesting approach to overcome drug resistance to erlotinib treatment in NSCLC.

## 1. Introduction

Non-small cell lung cancers (NSCLCs) harboring activating mutations in the epidermal growth factor receptor (EGFR) tyrosine kinase domain are typically treated with EGFR tyrosine kinase inhibitors [1]. However, the vast majority of these tumors develop drug resistance within several months. A broad spectrum of resistance mechanisms has been identified that includes the acquisition of secondary mutations in the EGFR; activation of bypassing signals by the amplification of EGFR-related family members, such as the human epidermal growth factor (HER)-2 or the unrelated c-Met; phenotypic transformation; or the activation of downstream signaling molecules [2,3,4].

While next generation sequencing has enabled deep insights into the genetic alterations of tumor cells, nongenetic alterations driven by posttranslational modification or changes in protein stability may also be relevant for tumor progression and drug resistance yet are far less accessible than genetic alterations. Signaling of the EGFR receptor can be imagined as a bow-tied system of network signaling, consisting of a broad ligand input module, including the EGFR-related members of the HER family; an output module that consists of multiple signaling pathways; and a core process that, by biochemical interactions of adaptor proteins with the receptor, tightly regulates the signaling network [5]. In order to identify changes within this interactome of adaptor proteins at the EGFR receptor, we have developed a method based on affinity purification of the receptor and high-resolution mass spectrometry [6]. We applied this technique to NSCLC cells adapted to grow in the presence of EGFR tyrosine kinase inhibitors as a cellular model of acquired drug resistance and found a constitutive activation of Akt in a subline of the NSCLC cell line HCC4006 adapted to the EGFR tyrosine kinase inhibitor erlotinib (HCC4006^r^Erlo^0.5^) [7]. 

Here, we investigated the molecular mechanism that caused constitutive Akt activation and strategies to revert drug resistance. While cell proliferation regulating inhibitor of phosphatase 2A (CIP2A) was downregulated upon EGFR inhibition by erlotinib in parental HCC4006 cells, it was constitutively present in HCC4006^r^Erlo^0.5^ cells. Pharmacological repression of CIP2A in HCC4006rErlo0.5 cells by bortezomib reactivated protein phosphatase 2A, which resulted in dephosphorylation and inactivation of Akt signaling. Furthermore, p21 and p27 were induced, and cells were arrested in cell cycle phase G2/M. Quantitative pharmacodynamics interaction modeling revealed antagonistic interactions between erlotinib and bortezomib at concentrations higher than 100 nM in HCC4006 cells and a higher potency of bortezomib in HCC4006^r^Erlo^0.5^ cells without any combinatory effect, suggesting a switch in oncogene addiction.

## 2. Materials and Methods

### 2.1. Chemicals

All chemicals were of analytical grade. Ribonuclease A, propidium iodide, and phosphate buffered saline (PBS) were purchased from Sigma–Aldrich Chemie GmbH (Munich, Germany). Inhibitors erlotinib and MK2206 were supplied by Selleckchem (Munich, Germany), and bortezomib was supplied by Cell Signaling Technology (New England Biolabs GmbH, Frankfurt/Main, Germany). 

Primary antibodies against pEGFR (Phospho-EGF Receptor (Tyr1068) (D7A5) XP^®®^ Rabbit mAb), EGFR (EGF Receptor (D38B1) XP^®®^ Rabbit mAb), pAkt (Phospho-Akt (Ser473) (D9E) XP^®®^ Rabbit mAb), Akt (Akt Rabbit pAb), pMAPK (Phospho-p44/42 MAPK (Erk1/2) (Thr202/Tyr204) (E10) Mouse mAb), MAPK (p44/42 MAPK (Erk1/2) (3A7) Mouse mAb), pSTAT3 (Phospho-Stat3 (Tyr705) (D3A7) XP^®®^ Rabbit mAb), STAT3 (Stat3 (79D7) Rabbit mAb), p53 (1C12 Mouse mAb), as well as antimouse (horse radish peroxixdase (HRP)-linked goat IgG) and antirabbit (HRP-linked goat IgG) secondary antibodies were obtained from Cell Signaling Technology (New England Biolabs GmbH, Frankfurt/Main, Germany). Antibodies for CIP2A (2G10-3B5, mouse monoclonal IgG2b), p-PP2A (p-PP2A-Cα/β Rabbit pAb (Tyr 307)), and dem-PP2A (demethylated PP2A-C (4B7), Mouse mAb) were purchased from Santa Cruz Biotechnology, Inc. (Dallas, Texas, USA). p27 (Anti-p27 KIP 1 Rabbit mAb [Y236]), phospho-p27 (Recombinant Anti-p27 KIP 1 (phospho S10) antibody (EP233(2)Y)), and p21 (Anti-p21 Rabbit mAb (EPR3993)) antibodies were obtained from abcam (Cambridge, MA, USA). Anti-Glycerinaldehyd-3-Phosphat-Dehydrogenase (GAPDH) Mouse mAb (H86504M) was obtained from Meridian Life Science,^®®^ Inc., (Memphis, TN, USA).

### 2.2. Cell Culture

The NSCLC cell line HCC4006 (HCC) was purchased from ATCC (Manassas, VA, USA). The subline HCC4006^r^ERLO^0.5^, with acquired resistance to erlotinib, was established as previously described [8] and derived from the Resistant Cancer Cell Line (RCCL) collection (https://research.kent.ac.uk/industrial-biotechnology-centre/the-resistant-cancer-cell-line-rccl-collection/; last access 19 March 2021) [9]. All cell lines were cultured in DMEM/HAM’s F12 medium (Sigma–Aldrich Chemie GmbH, Munich, Germany) supplemented with 10% fetal bovine serum (Gibco by Thermo Fisher Scientific, BV & Co KG, Braunschweig, Germany) at 37 °C in a humidified 5% CO_2_. Medium of HCC4006rERLO0.5 cells was additionally supplemented with 0.5 µM erlotinib.

### 2.3. Western Immunoblotting

HCC4006 and their subline HCC4006^r^ERLO^0.5^, adapted to grow in the presence of 0.5 µM erlotinib, were incubated with inhibitors MK2206 (Akt) and bortezomib (CIP2A), and resulting effects on downstream signaling proteins were assessed by SDS-PAGE and Western Blotting. Cells were plated in 6-well plates at 2 × 10^5^ cells/well for HCC4006 and 5 × 10^5^ cells/well for HCC4006^r^ERLO^0.5^. After overnight adhesion, cells were incubated as indicated, washed with ice cold PBS, and each sample lysed with 100 µl lysis buffer (50 mM Tris pH 7.6, 100 mM NaCl, 5 mM EDTA, 0.2 mM sodium vanadate, 0.1 % Triton^®®^ X-100). We separated 50 μg of total protein on 10% SDS-polyacrylamide gels, and protein was transferred to nitrocellulose membranes through electroblotting (6 mA/cm^2^) for 1.5 h. Membranes were incubated with 10% milk powder in TBST buffer (Tris 2.42 g/L, NaCl 8.5 g/L, Tween-20 0.05%) as blocking reagent (2 h or overnight at 4 °C). Primary antibodies were incubated overnight at 4 °C and used in a dilution of 1:1000, except anti-EGFR-antibody, which was used in a 1:3000 dilution. Secondary HRP-conjugated antibodies were incubated in a dilution of 1:3000 for 1 h at room temperature. Chemiluminescence signals were developed with Immunstar Western CTM (BioRad, Munich, Germany), and specific bands were visualized by ChemoCam imaging system (Intas, Göttingen, Germany).

### 2.4. Cell Proliferation Analysis

Cells were seeded into 96-well plates (40,000 cells/well for HCC4006 and 80,000 cells/well for HCC4006^r^ERLO^0.5^) and incubated with inhibitors solubilized in a maximum of 0.5 µM DMSO for 72 h. Cell viability was assessed by measuring transformation of the formazan derivative 3-(4,5-dimethylthiazole-2-yl)-2,5-diphenyltetrazoliumbromide (MTT) by mitochondrial NADPH at 37 °C for 4 h at 570 nm (SpectraMax Plus 384 Microplate Reader, Molecular Devices, Sunnyvale, CA, USA) and normalized to the DMSO solvent control after background subtraction.

### 2.5. Cell Cycle Analysis

For cell cycle analysis, cells were trypsinized and 0.5 × 10^6^ cells per sample fixed and permeabilized in 1 mL ice-cold ethanol (70%) at least 1 h at 4 °C. After fixation, the cells were centrifuged for 5 min at 150× *g*, washed with PBS, and incubated with 25 µL RNase A (1 mg/mL; Life Technologies, Carlsbad, CA, USA) in 500 µL PBS for 30 min at 37 °C. Following further centrifugation and washing steps, cells were carefully resuspended in 500 µL FACS buffer (MACSQuant Running Buffer, Miltenyi Biotec, Bergisch Gladbach, Germany) and stained with 25 µL propidium iodide (1 mg/mL in PBS, Sigma–Aldrich, Steinheim, Germany) with gentle vortexing. Samples of 5000 cells were analyzed using flow cytometry (MacsQuant, Miltenyi Biotec, Bergisch Gladbach, Germany).

### 2.6. Genetic Mutation Analysis

Genomic DNA was isolated using the MagNA Pure 24 total NA isolation kit (Roche Molecular Systems, Inc.), as described by the manufacturer’s protocol. DNA library of genes of interest were prepared using the TruSight Tumor 15 kit (Illumina, Inc., San Diego, CA, USA) and sequenced using a MiniSeq Rapid High output Reagent Kit on a MiniSeq System (Illumina, Inc.). Sequence analysis was performed using Illumina BaseSpace Variant Interpreter with the BaseSpace Annotation engine 1.6.2.0 based on the human hg19 reference genome (Illumina, Inc.).

### 2.7. Data Analysis

Combination Index according to Chou–Talalay

In order to quantify combination effects, the combination index theory of Ting-Chao Chou and Paul Talalay was used [10], which is based on the concentration additivity concept of Loewe et al. [11]. In brief, the combination index was calculated using the following equation:

(1) $$\mathrm{CI}\;=\;\frac{{\left(D\right)}_{1}}{{\left(D_{x}\right)}_{1}}+\frac{{\left(D\right)}_{2}}{{\left(D_{x}\right)}_{2}}$$

Therein, (D)_1_ and (D)_2_ describe doses or concentrations of drugs to result in a specific effect x when applied in combination. The contribution of the single compounds to the combination effect is deduced from the combination ratio as:

$${\left(D\right)}_{1}\;=\;(D_{x}{)}_{1,2}\cdot \frac{P}{\left(P+Q\right)}\;\;{\left(D\right)}_{2}\;=\;(D_{x}{)}_{1,2}\cdot \frac{Q}{\left(P+Q\right)},$$

where P and Q describe the relative amount of the drugs in the drug combination and (Dx)_1,2_ the combination dose to result in a specific effect x. (Dx)_1_ and (Dx)_2_ in Equation (1) describe doses or concentrations of the drugs to result in a specific effect x when applied as single agents. These values can be calculated from the transformed median effect equation

$$D_{x}\;=\;D_{m}\cdot {\left(\frac{f_{a}}{f_{u}}\right)}^{\frac{1}{m}}$$

where D_m_ describes the median effect dose, f_a_ the affected fraction, f_u_ the unaffected fraction, and m the shape of the dose effect curve.

The combination index was calculated using the software CompuSyn (ComboSyn Inc. Paramus, NJ, USA).

### 2.8. GPDI-Model Structure

The General PharmacoDynamic Interaction (GDPI) Model [12] was used for quantitative description of the interactions of combined inhibitor exposure. Briefly, this model combines the mechanistic description of sigmoid E_max_-type models with the additive components of Loewe Additivity (LA) or Bliss Independence (BI) models. An interaction parameter is implemented on either the maximal effect, Emax, or the concentration of 0.5 × E_max_, EC_50_, (or, in rare cases, on both), allowing quantitative interpretation of the interaction. This interaction parameter is dependent on the perpetrator concentration, allowing for a dynamic rather than a static interaction. Equation (2) shows an example using BI, based GPDI implemented on maximum effect:

(2) $$E_{AB}=E_{A}+\;E_{B}-\;E_{A}*E_{B}$$

$$E_{A}=\;\frac{E_{max,A}*\;\left(1+\;\frac{INT_{AB}*C_{B}}{EC_{50,INT,AB}+\;C_{B}}\right)*C_{A}^{H_{A}}}{EC_{50,A}^{H_{A}}+\;C_{A}^{H_{A}}}\;E_{B}=\;\frac{E_{max,B}*\;\left(1+\;\frac{INT_{BA}*C_{A}}{EC_{50,INT,BA}+\;C_{A}}\right)*C_{B}^{H_{B}}}{EC_{50,B}^{H_{B}}+\;C_{B}^{H_{B}}}$$

In cases of sparse or less informative data, several simplifications of the model structure are possible. Firstly, a joint interaction parameter can be estimated (IntAB = IntBA), assuming the nature of the interaction to affect both drugs similarly. Secondly, the interaction EC_50_ can be assumed equal to the monotherapy EC_50_ (EC_50Int,AB_ = EC_50B_ and EC_50Int,BA_ = EC_50A_), assuming the nature of the interaction to be similar to the nature of the drug effect. Lastly, the structural model can be simplified from a sigmoidal model to a linear or constant effect model in some cases.

### 2.9. Model Estimation and Evaluation Tools

Several model evaluation tools were used in this work to choose the best model and assess its quality. Parameter estimation was performed by maximation of the log likelihood, which was approached using minimization of the extended least squares error (ELS) with a combined error model [13] (Equation (3)):

(3) ELS = 0.5 ∗∑i=1n (PREDi − OBSi^2σ2 + logσ2)

$$SIG2_{PROP}\;=\;prop\;error*PRED_{i}^{2}\;SIG2_{ADD}\;=\;add\;error\;\sigma^{2}\;=\;{\left(SIG2_{ADD}\;+\;SIG2_{PROP}\right)}^{2}$$

with PRED_i_ as the ith prediction, OBS_i_ the ith experimental observation, and n the number of experimental observations. This error was minimized using a sequential application of a robust Nelder–Mead algorithm, followed by a conjugate gradient method for refinement. Models were evaluated by the precision of their parameter estimates, calculated from the inverse of the square root of the diagonal elements of the Fisher Information matrix, calculated from the Hessian. To perform model selection, models with sufficient parameter precision were compared based on the Akaike Information Criterion (AIC), which favors parsimony by including a penalty term for the number of parameters (k):

(4) $$AIC=\min\left(ELS\right)+2*k$$

Finally, selected models were compared to the experimental data for deviations, where more than 15% deviation from observed effect or no overlap with the 95% confidence interval of the t-distribution estimated form the experimental observations were considered significant deviations. The entire model selection and evaluation workflow was established using R (v. 3.4.4) and RStudio (v. 1.1.447).

### 2.10. Live Cell Imaging

Cells were cultured in 35 mm µ-cell culture plates (Ibidi, Planegg, Germany) in DMEM/F12-Ham medium by plating 300,000 cells per well and controlled for confluency of 60–70%. Prior to the experiment, cells were washed with PBS, and medium was replaced with fresh medium containing 10 nM bortezomib. The plates were then immediately placed under the time lapse-microscope (Biostation II, Nikon Instruments, Düsseldorf, Germany). During the experiment, the incubation chamber was kept at 37 °C and 5% CO_2_ atmosphere. Images were taken every 3 min for 72 h and converted into time lapse movies of 90 s duration. 

### 2.11. Colony Formation Assay

We treated 1 × 10^5^ cells with either 10 nM bortezomib or DMSO as control for 24 h. Cells were then washed with PBS, trypsinized to produce a single-cell suspension, and counted. Then, 5 × 10^3^ cells were plated on a 150 × 20 mm petri dish and grown for 2 weeks. The medium was replaced every 3 days. After 2 weeks, the cells were fixed and stained with a 1% formaldehyde 0.05% (m/v) crystal violet solution for 20 min at room temperature. After extensive washing with deionized water, the plates were imaged and the colonies counted manually. One colony was defined as >50 cells. The surviving fraction (SF) was calculated as

SF = (no. of colonies formed after treatment)/(no. of cells seeded × PE)(5)

with the plating efficiency (PE, %) calculated from

PE = (no. of colonies formed)/(no. of cells seeded) × 100

### 2.12. Statistical Analysis

All experiments were carried out in triplicates. Data were analyzed for statistical differences using student’s t-test for single comparisons and one-way ANOVA analyses for multiple comparisons on one dataset. Tests were performed using the GraphPad Prism Version 5.02 software (GraphPad Software, Inc., San Diego, CA, USA).

## 3. Results

### 3.1. The CIP2A/PP2A/Akt Signaling Module Is Constitutively Active in HCC4006^r^Erlo^0.5^ Cells

**Figure 1:**
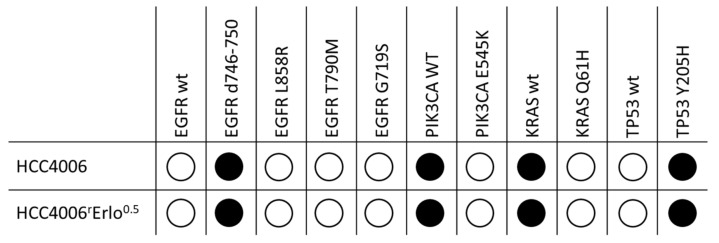
Genetic characteristics of native HCC4006 and HCC4006^r^Erlo^0.5^ cells adapted to grow in the presence of 0.5 µM erlotinib. DNA was isolated, and a DNA library of fifteen common tumor genes was prepared using the TrusightTumor15 panel and analyzed on a MiniSeq System using Illumina BaseSpace Variant Interpreter and BaseSpace Annotation Engine 1.6.2.0 (Illumina, Inc., San Diego, CA, USA). Black bullets denote the identified genetic variant of the respective gene.

We initially screened fifteen common tumor genes for secondary mutations, which may cause constitutive Akt activation, using next generation sequencing. The investigated tumor genes were AKT1, BRAF, EGFR, ERBB2, FOXL2, GNA11, GNAQ, KIT, KRAS, MET, NRAS, PDGFRA, PIK3CA, RET, and TP53. While the original mutations present in the HCC4006 cells (EGFR d746-750, TP53 Y205H) were also found in the adapted HCC4006^r^Erlo^0.5^ cell line, no secondary mutation was found in the investigated tumor oncogenes (Figure 1).

**Figure 2:**
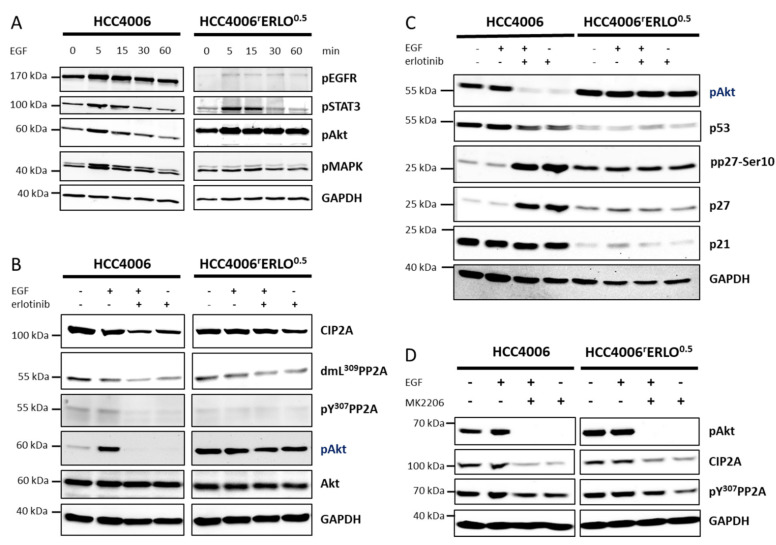
Akt signaling is constitutively active in HCC4006^r^Erlo^0.5^ cells adapted to grow in the presence of 0.5 µM erlotinib. Cells were (**A**) incubated with 100 ng/mL epidermal growth factor (EGF) for the depicted times, (**B**,**C**) preincubated with 1 µM erlotinib for 24 h before adding 100 ng/mL EGF for another 30 min, or (**D**) preincubated with 20 µM MK2206 for 24 h before adding 100 ng/mL EGF for another 30 min. Proteins were separated on a 10% polyacrylamide gel and blotted on a nitrocellulose membrane, and specific target proteins were detected using target specific primary and species-specific secondary antibodies, coupled with horseradish peroxidase, as listed in the methods section. Signals were developed using a chemoluminescence substrate and detected with the ChemoCam System (Intas, Göttingen, Germany). A representative blot of at least three biological replicates is shown.

To further characterize the constitutive activation of Akt, the cells were stimulated with epidermal growth factor (EGF) and the EGFR signaling activity, represented by the STAT-, Akt, and MAPK pathways, was followed over time. In HCC4006 cells, EGF-induced EGFR signaling peaked at 5 min after stimulation and returned to the basal level after 60 min. A similar signaling kinetic was observed in HCC4006^r^Erlo^0.5^ cells for all observed EGFR signaling pathways, except for the Akt signaling. In HCC4006^r^Erlo^0.5^ cells, Akt signaling was constitutively activated and not affected by upstream receptor signaling (Figure 2A). 

Cell proliferation regulating inhibitor of phosphatase 2A (CIP2A) has been shown to activate Akt signaling via inhibition of Akt dephosphorylation in different types of cancer, including lung cancer [14,15,16,17,18]. Therefore, we investigated protein levels of CIP2A in response to stimulation or inhibition of EGFR activity in HCC4006 and HCC4006^r^Erlo^0.5^ cells. In HCC4006 cells, EGF stimulation did not affect CIP2A levels, but EGFR inhibition by erlotinib markedly reduced the cellular CIP2A amount. Reduced CIP2A levels resulted in protein phosphatase 2A (PP2A) activation, as indicated by reduced phosphorylation of a tyrosine in position 307 of the amino acid sequence and demethylation of a lysine in position 309, followed by an almost complete loss of Akt phosphorylation. In HCC4006^r^Erlo^0.5^ cells, neither EGF nor erlotinib affected CIP2A protein levels or PP2A activity (Figure 2B, Supplementary Figure S1).

Similar effects were observed when downstream substrates of Akt were investigated. In HCC4006 cells, EGFR inhibition resulted in a slight decrease of p53 and a robust increase of the cell cycle regulator p27. In HCC4006^r^Erlo^0.5^ cells, these proteins were completely unaffected (Figure 2C). Finally, we observed a feedback regulation in both of the cell lines in that inhibition of Akt activity resulted in a reduction of CIP2A protein and a reduction of the PP2A inactivity marker pY307 (Figure 2D).

### 3.2. Bortezomib Reduces Cellular CIP2A Levels, Inhibits Akt Phosphorylation, and Induces p21 Expression and Cell Cycle Arrest in G2/M Phase

**Figure 3:**
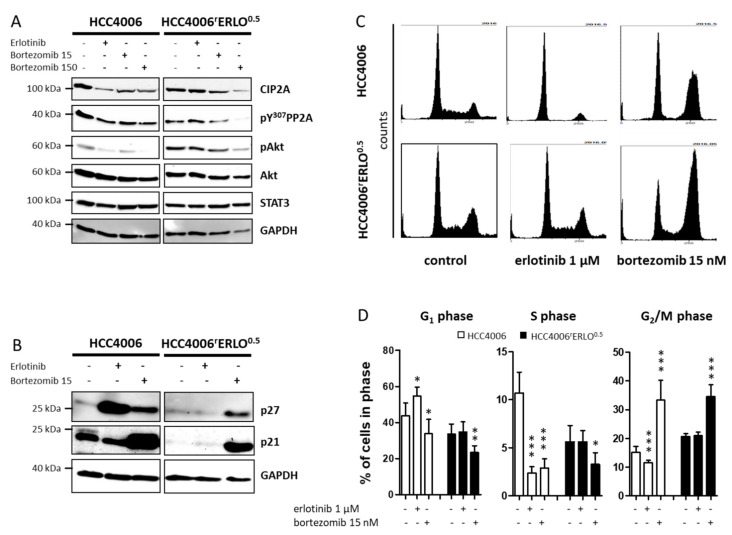
Bortezomib downmodulates cell proliferation regulating inhibitor of phosphatase 2A (CIP2A), restores regulation of Akt, and arrests cells in the G_2_/M phase of the cell cycle. Cells were incubated with 1 µM erlotinib, 15 nM bortezomib (**A**,**B**) or 150 nM bortezomib (**A**) for 24 h. Proteins were separated on a 10% polyacrylamide gel and blotted on a nitrocellulose membrane, and specific target proteins were detected using target specific primary and species-specific secondary antibodies, coupled with horseradish peroxidase, as listed in the methods section. Signals were developed using a chemoluminescence substrate and detected with the ChemoCam System (Intas, Göttingen, Germany). A representative blot of at least three biological replicates is shown. (**C**) Distribution of the cell cycle phases was analyzed by propidium iodide staining of fixed cells and detection of the fluorescence intensities using a MACSQuant flow cytometer after incubating the cells with 1 µM erlotinib or 15 nM bortezomib for 24 h. (**D**) The relative amount of cells in each phase of the cell cycle is displayed as mean ± SD, *n* = 6, * *p* < 0.05, *** *p* < 0.001.

The proteasome inhibitor bortezomib has been shown to reduce cellular CIP2A protein levels independently of its proteasome inhibition activity [14,15]. Bortezomib reduced CIP2A protein levels at concentrations of 15 nM and 150 nM with accompanying reduction of PP2A inactivity marker pY307 and a reduction of pAkt in both HCC4006 and HCC4006^r^Erlo^0.5^ cell lines (Figure 3A, Supplementary Figure S2). Similarly, bortezomib markedly induced p27 and strongly induced p21 in both cells lines (Figure 3B), which was in line with a significant induction of cells arrested in the G_2_/M phase (33.3 ± 6.9% of cells in G_2_/M with 15 nM bortezomib vs. 15.2 ± 1.9% at control conditions for HCC4006, *n* = 7, *p* < 0.0001 and 34.5 ± 4.1% vs. 20.6 ± 1.1%, respectively for HCC4006^r^Erlo^0.5^, *n* = 6, *p* < 0.0001; one-way ANOVA with Dunnett’s Multiple Comparison Test; Figure 3C,D). 

### 3.3. Combination Analyses Reveal Antagonistic Effects of Erlotinib and Bortezomib in HCC4006 Cells and Sensitization to Bortezomib in HCC4006^r^Erlo^0.5^ Cells

First, combination effects were analyzed calculating the combination index, according to the method of Chou-Talalay. This method is based on the concentration additivity concept developed by Loewe et al. and proposes additive effects of substances given similar targets and mode of actions [10]. 

**Figure 4:**
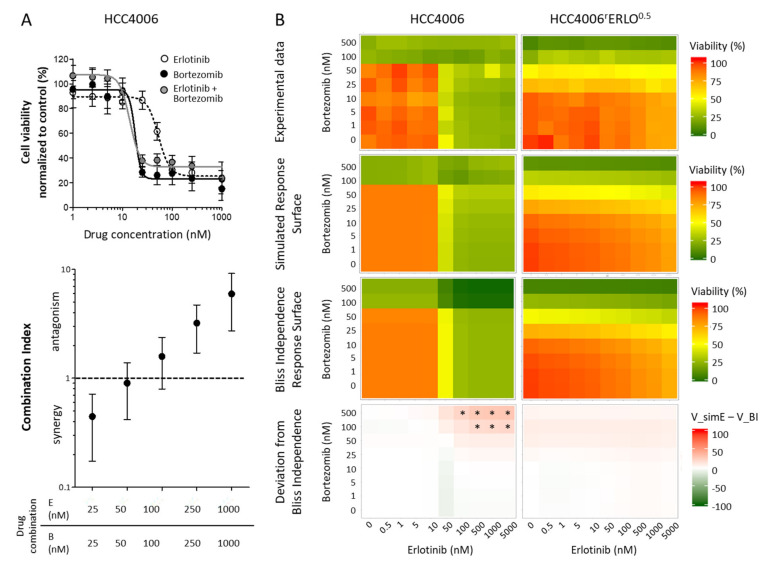
Combination effects of erlotinib and bortezomib in HCC4006rErlo0.5 cells do not deviate from additivity. (**A**) Viability of HCC4006 cells was assessed for combinations of equimolar concentrations of erlotinib (E) and bortezomib (B) and combination index was calculated as explained in the methods section; values represent mean ± SD, *n* = 3. (**B**) Viability of HCC4006 and HCC4006^r^Erlo^0.5^ cells was assessed in a checkerboard format. Values represent means of three independent experiments. Simulation was conducted as described in the methods section, * *p* < 0.05.

Any deviation from additivity suggests interacting effects of the two substances. Calculation of the combination indices revealed nonstatistical trends of synergistic effects at an equimolar combination with 25 nM and antagonistic effects with 250 nM and 1000 nM of both drugs for HCC4006 cells (Figure 4A). As calculation of the combination index requires distinct IC50 values, this measure could not be calculated reliably for HCC4006^r^Erlo^0.5^ cells. Therefore, a GPDI Model [12] was used for quantitative description of the interactions (Figure 4B). In the sensitive HCC4006 cells, a full GPDI model on maximum effect performed best. An antagonistic interaction was quantified in both directions (of very similar magnitude), leading to deviations from BI when both erlotinib and bortezomib concentrations were >100 nM. In the resistant HCC4006^r^Erlo^0.5^ cells, no interaction parameters could be estimated precisely, indicating the lack of interaction. Indeed, a model assuming no significant interaction described the experimental data better than other GPDI model candidates. In addition, the effect of bortezomib in HCC4006^r^Erlo^0.5^ cells was increased, compared to the sensitive HCC4006 cells (E_max_ of 0.911 instead of 0.585, a 55.7% increase and EC_50_ of 52.4 nM, instead of 73.0 nM, a 28% decrease, Table 1).

**Figure 5:**
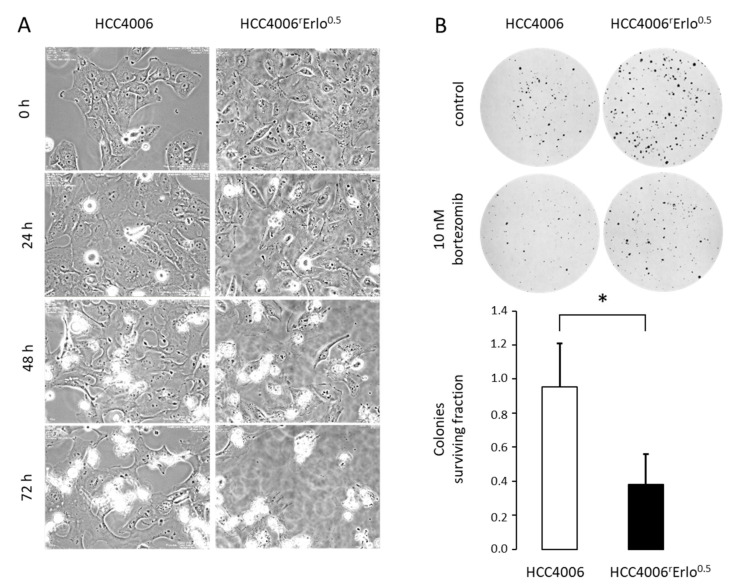
HCC4006^r^Erlo^0.5^ cells are more sensitive to bortezomib compared to their native HCC4006 cells. (**A**) HCC4006 and HCC4006^r^Erlo^0.5^ cells were cultivated in a time-lapse microscope (Biostation II, Nikon Instruments, Düsseldorf, Germany) at 37 °C and 5% CO_2_ atmosphere in the presence of 10 nM bortezomib. Representative images were taken at the indicated time points. (**B**) Cells were plated in 150 mm cell culture dishes, and colonies were allowed to grow for 14 days. After staining with crystal violet, colonies consisting of >50 cells were counted manually. The colonies surviving fraction was calculated as described in the methods section. Values represent mean ± SD, *n* = 3, * *p* < 0.05.

In order to confirm the hypersensitivity of HCC4006^r^Erlo^0.5^ cells to bortezomib observed in the model, we conducted live cell imaging of HCC4006 and HCC4006^r^Erlo^0.5^ cells in the presence of 10 nM bortezomib over a time period of 72 h. In the presence of 10 nM bortezomib HCC4006^r^Erlo^0.5^ cells, cell death is induced as soon as 30 hours after starting the bortezomib incubation. In contrast, in HCC4006 cells, the onset of cell death induction was delayed to approximately 40 hours, with a subsequent delay in cell death kinetics (Figure 5A, Supplementary Video). In order to quantify these effects, the impact of bortezomib on clonogenic growth was assessed. The colonies surviving fraction after an exposure to 10 nM bortezomib for two weeks were significantly reduced in HCC4006^r^Erlo^0.5^ cells, compared to HCC4006 cells (0.38 ± 0.18 vs. 0.95 ± 0.25, respectively, *p* < 0.05, Figure 5B).

## 4. Discussion

Exploring mechanisms of drug resistance to targeted small molecule drugs is critical for an extended clinical benefit in the treatment of non-small cell lung cancer (NSCLC) patients carrying activating EGFR mutations. Sequential targeted therapy is currently based on identifying secondary resistance mutations. However, in a substantial number of patients, no secondary mutation can be found. Moreover, nongenetic mechanisms cannot be detected. Here, we identified constitutive cell proliferation regulating inhibitor of protein phosphatase 2A (CIP2A) in the HCC4006^r^Erlo^0.5^ NSCLC cell line adapted to erlotinib as a model of acquired drug resistance. Constitutive CIP2A resulted in a constitutive activation of Akt signaling. The proteasome inhibitor bortezomib was able to reduce CIP2A levels which resulted in an activation of protein phosphatase 2A and deactivation of Akt. Furthermore, accumulation of the cell cycle inhibitors p21 and p27 was accompanied by a cell cycle arrest in the G_2_/M phase. Combination experiments with erlotinib and bortezomib revealed a lack of interaction between the two drugs. However, the effect size of bortezomib was higher in the adapted HCC4006^r^Erlo^0.5^ resistance cell model, compared to the erlotinib-sensitive HCC4006 cells, as indicated by an almost two-fold increase in E_max_ and almost one-third decrease in EC_50_ in the concentration–effect model; an earlier onset of cell death induction in live cell imaging experiments; and a reduced colony surviving fraction in a colony formation assay. 

CIP2A has been identified as a prognostic marker in non-small cell lung cancer, as its expression was higher in lung tumor tissue compared to corresponding normal tissue [19], and high levels of the protein in tumor samples were associated with reduced overall survival [19,20,21]. In vitro, the role of CIP2A has mostly been investigated in cell model systems that carry the wildtype EGFR. Knockdown of CIP2A resulted in a reduction of cell proliferation and clonogenic growth, an increased induction of apoptosis, and an increased sensitivity to cisplatin [22]. Furthermore, downmodulation of CIP2A has been shown to be a prerequisite for sensitivity of EGFR wildtype NSCLC cell lines to EGFR tyrosine kinase inhibitors erlotinib [18,23] or afatinib [24] but also to natural compounds amentoflavone [25] and polyphyllins [26,27]. In EGFR mutated cells that carry the secondary T790M mutation and are intrinsically resistant to gefinitib, CIP2A downmodulation was shown to play a critical role in the antitumor mode of action of the natural compounds oridonin [28] and cucurbitacin B [29]. However, the role of CIP2A in the acquired resistance to EGFR tyrosine kinase inhibitors in model systems carrying activating EGFR mutations has not been investigated so far.

Aside from changes in CIP2A protein stability in HCC4006^r^Erlo^0.5^ cells upon treatment with erlotinib, we observed changes that were independent of erlotinib treatment in HCC4006^r^Erlo^0.5^ cells, compared to the parental HCC4006 cells. These were reduced levels of p53 protein and a reduced basal- and ligand-induced EGFR activation. HCC4006 cells carry the p53 Y205H mutation, which is associated with a median 15% transcriptional activity, compared to wildtype p53 [30]. Downregulation of p53 has already been observed in the course of acquired resistance to EGFR targeting drugs in lung cancer cell lines, and reconstitution of p53 resulted in a resensitization of these cells to the drugs the cells were adapted to [31,32]. However, these were cell lines carrying both wildtype p53 and EGFR. As the HCC4006 carry both mutated p53 and EGFR, it is unlikely that reduced levels of transcriptionally rather inactive p53 plays a major role for the resistance phenotype in our HCC4006^r^Erlo^0.5^ cell line. Constitutive activation of the EGFR in HCC4006 is an expression of their oncogene addiction phenotype [33]. This is lost in the adapted HCC4006^r^Erlo^0.5^ cells, which can be a result of several mechanisms, such as changes in cell surface receptor density, receptor trafficking, or a reduction in ligand release and autocrine signaling. 

**Figure 6:**
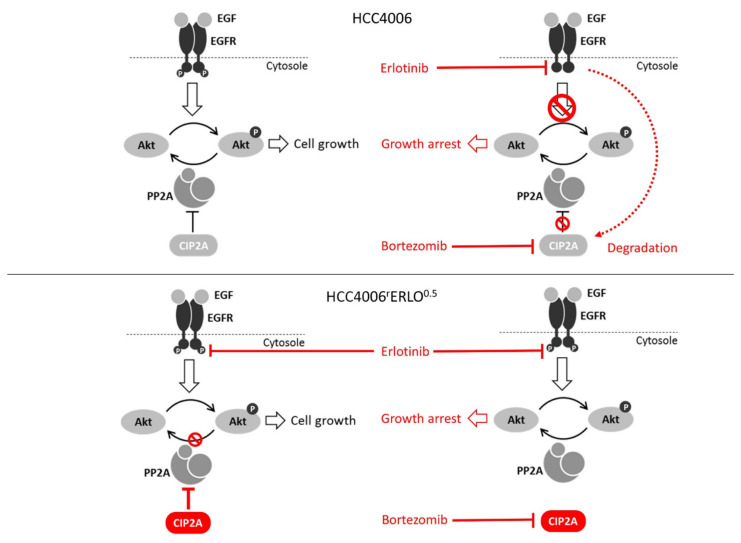
Schematic of the proposed mechanism of drug resistance of HCC4006rErlo0.5 cells developed against erlotinib.

The decrease in EGFR activity and the increase in responsiveness of HCC4006^r^Erlo^0.5^ cells to bortezomib, together without deviation from additivity in the combination treatment, suggests a switch in oncogene addiction from the EGFR to the Akt signaling pathway regulated by the constitutive presence of CIP2A (Figure 6, bottom left). By downmodulation of CIP2A, protein phosphatase 2A was reactivated to dephosphorylate Akt and to restore cell growth inhibition programs (Figure 6, bottom right). In contrast, HCC4006 cells sensitive to erlotinib growth inhibition responded to erlotinib by downmodulation of CIP2A (Figure 6, top right). 

This is somewhat unexpected, as downmodulation of CIP2A has been described in a rather treatment-unresponsive context so far. Interestingly, it was hypothesized recently that effective oncogenic kinase inhibition requires the simultaneous activation of the respective tumor suppressing phosphatase [34]. Our findings, thus, are well in line with this hypothesis. Further extensive time-resolved investigations are needed to dissect the interplay between EGFR receptor activation, signaling pathway activation, and signal regression, together with the translational or posttranslational modulation of CIP2A. 

Bortezomib is well-known as an inhibitor of the proteasome. In addition, there is evidence that CIP2A undergoes proteasomal degradation [35,36]. However, is has been shown in different model systems that the modulation of CIP2A induced by bortezomib is independent of its proteasome inhibition activity but rather depends on inhibition of transcriptional activity [37,38]. 

Sufficient activity of protein phosphatase 2A has been suggested as a general determinant of cancer drug response [34]. However, the mechanisms that lead to an impaired activity and, thus, cancer drug resistance, might vary substantially. Aside from CIP2A, other oncogenic PP2A inhibitor proteins have been identified, which all inhibit PP2A by different mechanisms [34]. In our cell system, we found CIP2A constitutively present in the adapted HCC4006^r^Erlo^0.5^ cells, escaping the downmodulating effect of erlotinib. Notwithstanding, the underlying mechanism is unknown and currently under investigation in our lab. We acknowledge that the findings reported here result from the investigation of a single cell line pair. An analysis of cell lines of the Genomics of Drug Sensitivity in Cancer database [39], however, revealed a significant correlation of CIP2A expression levels and the gefitinib and erlotinib sensitivity, which was significant for gefitinib when the analysis was restricted to lung cancer cell lines (Supplementary Figure S3). For erlotinib, the correlation was not significant in the lung cancer cell lines, which is likely due to the low number of lung cancer cell lines tested for erlotinib sensitivity. Taken together, these data further support a role of CIP2A in the response of lung cancer cells to EGFR tyrosine kinase inhibitors. 

A recent study using several sublines of a neuroblastoma cell line adapted to a small molecule revealed a broad range of resistance phenotypes, despite their initial limited intrinsic heterogeneity [40]. In non-small cell lung cancers (NSCLC) harboring EGFR mutations, the understanding of molecular and genetic intratumor heterogeneity has emerged as a pivotal task in the individualization process of cancer treatment [41]. Therefore, investigating the mechanisms of CIP2A stabilization in a range of NSCLC cell lines adapted to EGFR tyrosine kinase inhibitors is needed to contribute to a better understanding of clonal heterogeneity.

## 5. Conclusions

We have identified constitutive CIP2A in an EGFR-activating mutated cell model system of acquired resistance to the EGFR targeting the small molecule inhibitor erlotinib. Constitutive CIP2A could be downmodulated by the proteasome inhibitor bortezomib. The erlotinib-resistant cells were more sensitive towards bortezomib, compared to their erlotinib-sensitive parent cells. This suggests a switch in oncogene addiction and might provide a potential strategy to overcome erlotinib resistance in NSCLC.

## Figures and Tables

**Table 1:** Final General PharmacoDynamic Interaction (GPDI) model parameter estimates and their 95% confidence intervals (CI) for the interaction between erlotinib and bortezomib in the non-small cell lung cancer cell line HCC4006 and HCC4006^r^Erlo^0.5^ which has been adapted to the epidermal growth factor receptor (EGFR) tyrosine kinase inhibitor erlotinib. AIC: Akaike Information Criterion, EC_50_: Drug potency, the concentration at which half of the maximum effect is reached, E_max_: Maximum effect, H: Hill coefficient, sigmoidity of the concentration–effect relationship, GPDI_INT_: GPDI interaction parameter, maximal effect of perpetrator on victim, EC_50,INT_: Potency of the interaction, concentration of the perpetrator at which half of the maximum effect on the victim is reached, NA: not applied.

Parameters		HCC4006	HCC4006^r^Erlo^0.5^
AIC		−565	−408
Erlotinib	EC_50_ [nM] (95% CI)	35.2 (29.5–40.9)	3770 (−980–8514)
	E_max_ (95% CI)	0.586 (0.573–0.600)	0.426 (0.306–0.546)
	H (95% CI)	3.15 (1.90–4.41)	0.272 (0.157–0.387)
Bortezomib	EC_50_ [nM] (95% CI)	73.0 (60.4–111)	52.4 (46.1–58.8)
	E_max_ (95% CI)	0.585 (0.568–0.622)	0.911 (0.867–0.954)
	H (95% CI)	20.7 (−35.2–115)	1.47 (1.19–1.75)
Erlotinib effect on bortezomib E_max_	GDPI_INT_ (95% CI)	−0.492 (−0.595–−0.389)	0
	EC_50_,_INT_ [nM] (95% CI)	69.6 (38.6–101)	NA
Bortezomib effect on erlotinib E_max_	GDPI__INT_ (95% CI)	−0.546 (−0.713–−0.380)	0
	EC_50,INT_ [nM] (95% CI)	117 (64.5–170)	NA

## Data Availability

The data presented in this study are available in Supplementary Table S1.

## Supplementary Materials

The following are available online at https://www.mdpi.com/2073-4409/10/4/716/s1, Figure S1: Downmodulation of CIP2A by erlotinib is abrogated in HCC4006^r^Erlo^0.5^ cells; Figure S2: Bortezomib downmodulates CIP2A in HCC4006 and HCC4006rErlo0.5 cells and restores regulation of Akt signaling; Figure S3: Correlation of CIP2A expression levels and the gefitinb and erlotinib AUCs (areas under the curve) in the cell lines of the Genomcs of Drug Sensitivity in Cancer database; Table S1: Shared Data; Video S1A: Live cell time-lapse of HCC4006 incubated with 10 nM bortezomib for 72 h; Video S1B: Live cell time-lapse of HCC4006^r^Erlo^0.5^ incubated with 10 nM bortezomib for 72 h.
