# Supplementary material for: Constitutive Cell Proliferation Regulating Inhibitor of Protein Phosphatase 2A (CIP2A) Mediates Drug Resistance to Erlotinib in an EGFR Activating Mutated NSCLC Cell Line
Source: Cells. 2021 Mar 24;10(4):716. doi: 10.3390/cells10040716 (PMC8103245; doi:10.3390/cells10040716)
Supplement: Supplementary file 1 [file cells-10-00716-s001.zip › Ssupplementary Materials.docx]

Article

**Constitutive cell proliferation regulating inhibitor of protein phosphatase 2A (CIP2A) mediates drug resistance to erlotinib in an EGFR activating mutated NSCLC cell line**

Hisham Saafan ^1^, Ahmad Alahdab ^1^, Robin Michelet ^2^, Linus Gohlke ^1^, Janine Ziemann ^1,3^, Stefan Holdenrieder ^4^, Katie-May McLaughlin ^5^, Mark N. Wass ^5^, Jindrich Cinatl Jr ^6^, Martin Michaelis ^5^, Charlotte Kloft ^2^, and Christoph A Ritter ^1,*^

1 Institute of Pharmacy, Clinical Pharmacy, University of Greifswald, Friedrich-Ludwig-Jahn-Str. 17, 17489 Greifswald, Germany

2 Department of Clinical Pharmacy and Biochemistry, Institute of Pharmacy, Freie Universitaet Berlin, Germany

3 Central Unit for Infection Prevention and Control, University Medicine Greifswald, Greifswald, Germany

4 Institute of Laboratory Medicine, German Heart Center, Munich Technical University Munich

5 Industrial Biotechnology Centre, School of Biosciences, University of Kent, Canterbury, UK

6 Institute of Medical Virology, Goethe-University, Frankfurt am Main, Germany

* Correspondence: [ritter@uni-greifswald.de](mailto:ritter@uni-greifswald.de)

**Supplemetary Metarial**

 
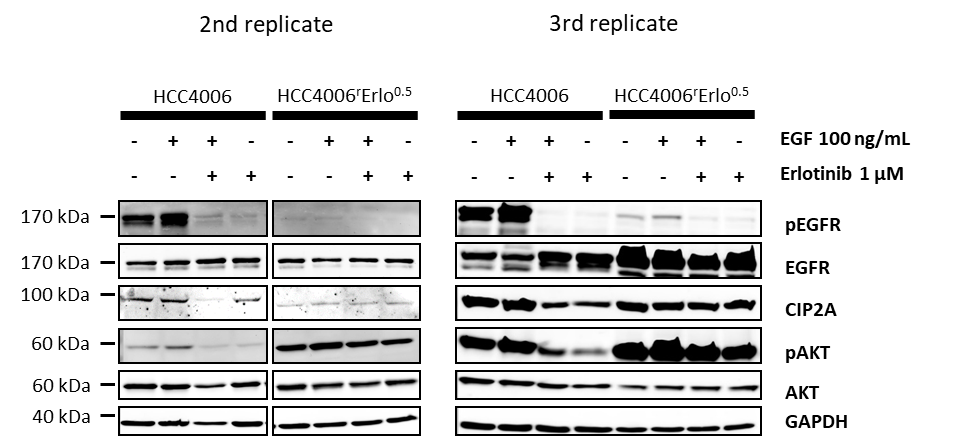


Figure S1: Down modulation of CIP2A by erlotinib is abrogated in HCC4006^r^Erlo^0.5^ cells.


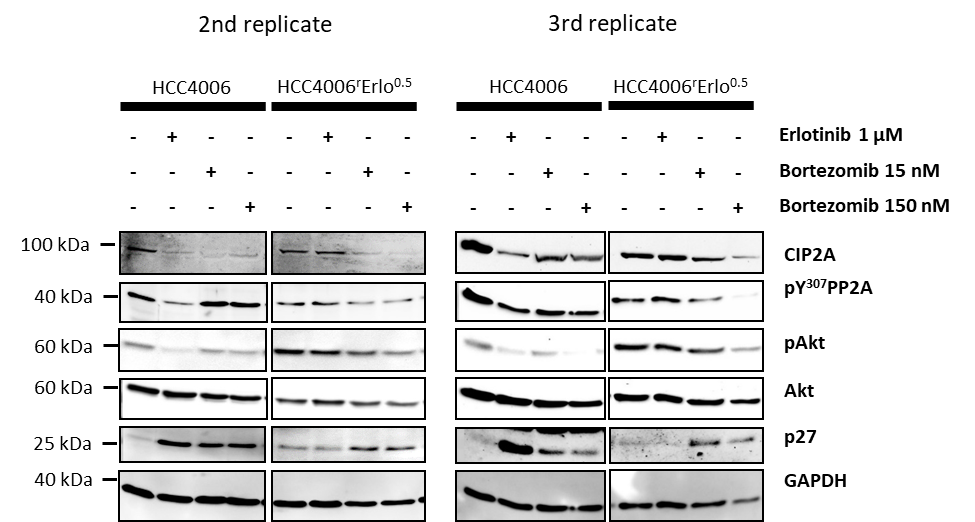


Figure S2: Bortezomib down modulates CIP2A in HCC4006 and HCC4006^r^Erlo^0.5^ cells and restores regulation of Akt signaling.

 
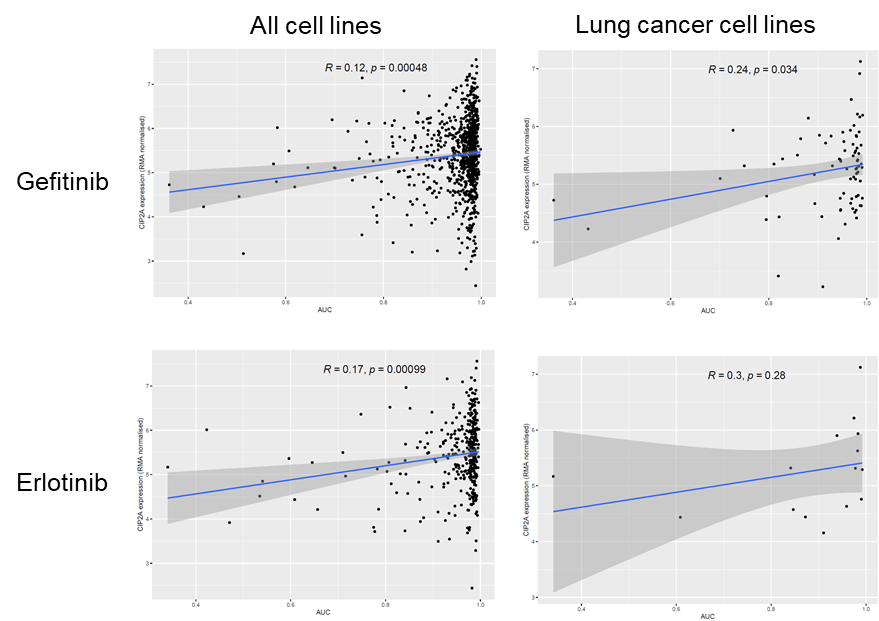


Figure S3: Correlation of CIP2A expression levels and the gefitinib and erlotinib AUCs (areas under the curve) in the cell lines of the Genomics of Drug Sensitivity in Cancer database.

Cell line drug sensitivity data (area under the curve, AUC) and RMA-normalised gene expression values were obtained from the Genomics of Drug Sensitivity in Cancer database (GDSC, 2016 release, https://www.cancerrxgene.org) [1]. Pearson correlation coefficients for the association between CIP2A expression and response to erlotinib and gefitinib were calculated using the cor.test function in R. Analyses were performed across all cancer cell lines (n=371 treated with erlotinib; n=848 treated with gefitinib) and across lung cancer cell lines (n=15 treated with erlotinib; n=77 treated with gefitinib). Scatterplots were generated using the ggplot2 R package.

1. Yang W, Soares J, Greninger P, Edelman EJ, Lightfoot H, Forbes S, Bindal N, Beare D, Smith JA, Thompson IR, Ramaswamy S, Futreal PA, Haber DA, Stratton MR, Benes C, McDermott U, Garnett MJ. Genomics of Drug Sensitivity in Cancer (GDSC): a resource for therapeutic biomarker discovery in cancer cells. Nucleic Acids Res. 2013 Jan;41(Database issue):D955-61. doi: 10.1093/nar/gks1111.

Video S1A: Live cell time-lapse of HCC4006 incubated with 10 nM bortezomib for 72 h;

Video S1B: Live cell time-lapse of HCC4006rErlo0.5 incubated with 10 nM bortezomib for 72 h.
