# Supplementary figures and images for: Constitutive Cell Proliferation Regulating Inhibitor of Protein Phosphatase 2A (CIP2A) Mediates Drug Resistance to Erlotinib in an EGFR Activating Mutated NSCLC Cell Line
Source: Cells. 2021 Mar 24;10(4):716. doi: 10.3390/cells10040716 (PMC8103245; doi:10.3390/cells10040716)

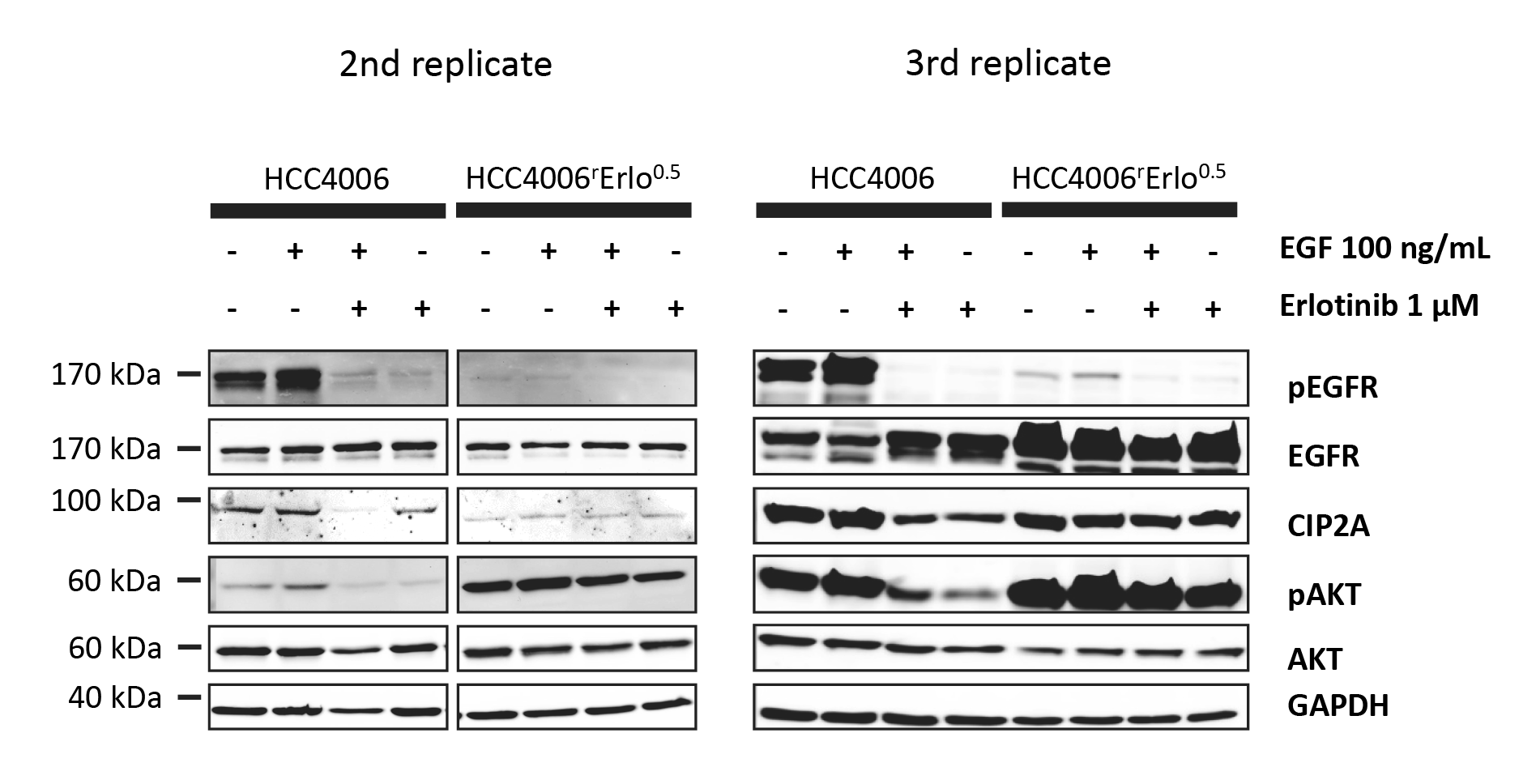

Supplement: Supplementary file 1 [file cells-10-00716-s001.zip › Supplementary Figure S1.tif]

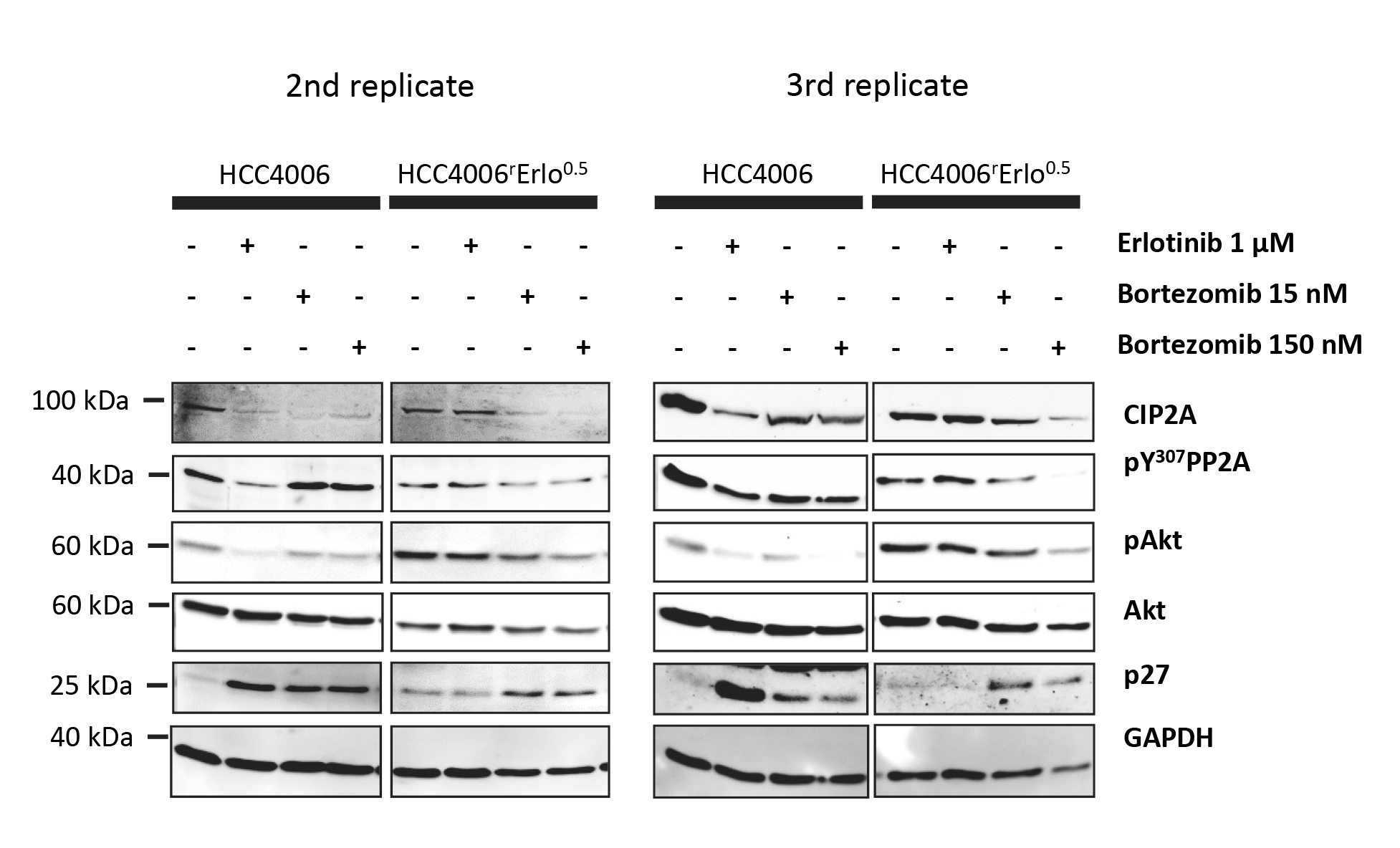

Supplement: Supplementary file 1 [file cells-10-00716-s001.zip › Supplementary Figure S2.tif]

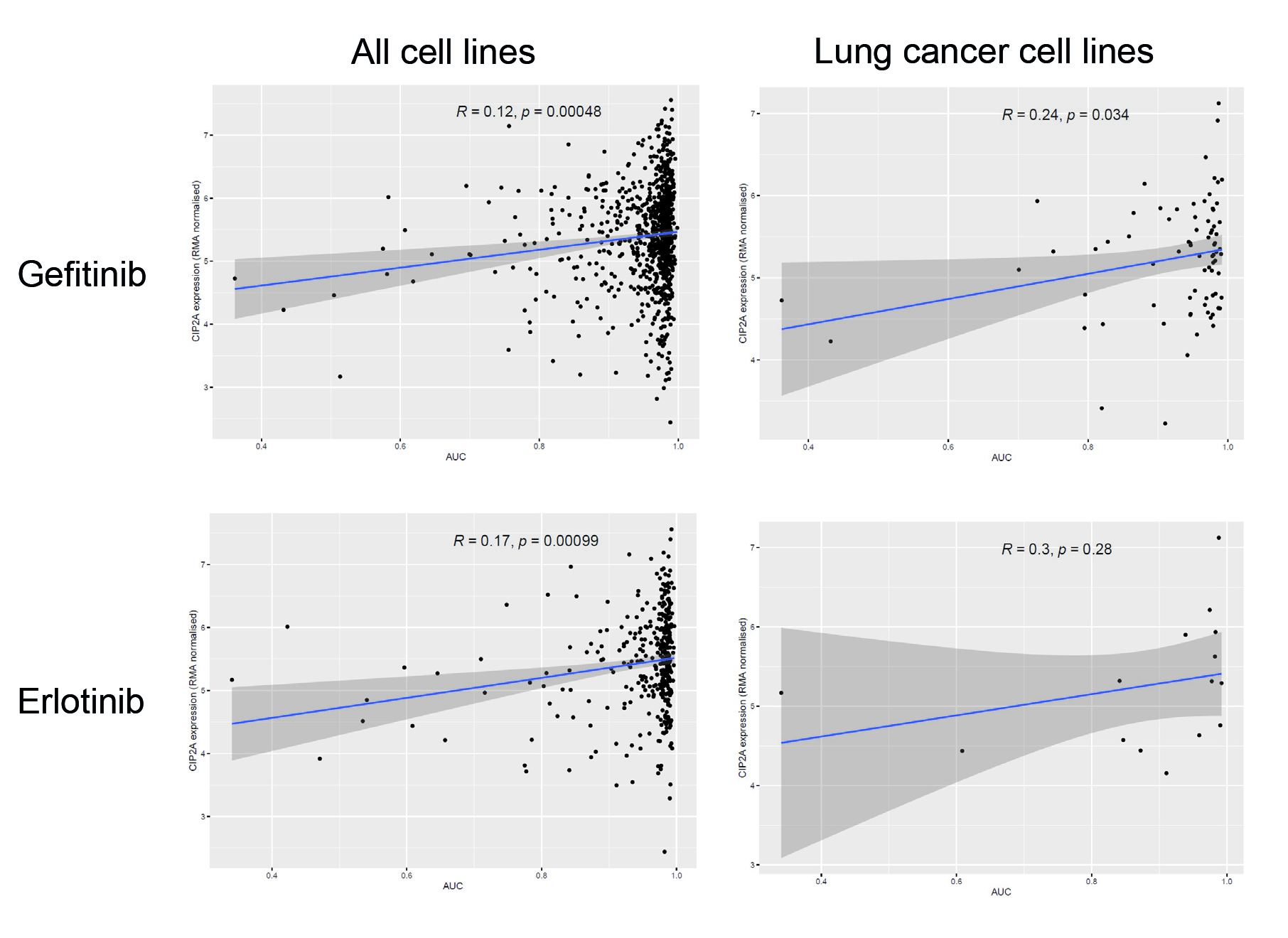

Supplement: Supplementary file 1 [file cells-10-00716-s001.zip › Supplementary Figure S3.tif]
